# Current Screening Methodologies in Drug Discovery for Selected Human Diseases

**DOI:** 10.3390/md16080279

**Published:** 2018-08-14

**Authors:** Olga Maria Lage, María C. Ramos, Rita Calisto, Eduarda Almeida, Vitor Vasconcelos, Francisca Vicente

**Affiliations:** 1 Departamento de Biologia, Faculdade de Ciências, Universidade do Porto, Rua do Campo Alegre s/nº 4169-007 Porto, Portugal; ritaisilc@gmail.com (R.C.); eduardamarqalmeida@gmail.com (E.A.); vmvascon@fc.up.pt (V.V.); 2 CIIMAR/CIMAR–Centro Interdisciplinar de Investigação Marinha e Ambiental–Universidade do Porto, Terminal de Cruzeiros do Porto de Leixões, Avenida General Norton de Matos, S/N, 4450-208 Matosinhos, Portugal; 3 Fundación MEDINA, Centro de Excelencia en Investigación de Medicamentos Innovadores en Andalucía, Parque Tecnológico de Ciencias de la Salud, 18016 Granada, Spain; carmen.ramos@medinaandalucia.es (C.R.); francisca.vicente@medinaandalucia.es (F.V.)

**Keywords:** anti-infectives, anticancer, neuroprotectors, marine natural products, drug discovery future trends

## Abstract

The increase of many deadly diseases like infections by multidrug-resistant bacteria implies re-inventing the wheel on drug discovery. A better comprehension of the metabolisms and regulation of diseases, the increase in knowledge based on the study of disease-born microorganisms’ genomes, the development of more representative disease models and improvement of techniques, technologies, and computation applied to biology are advances that will foster drug discovery in upcoming years. In this paper, several aspects of current methodologies for drug discovery of antibacterial and antifungals, anti-tropical diseases, antibiofilm and antiquorum sensing, anticancer and neuroprotectors are considered. For drug discovery, two different complementary approaches can be applied: classical pharmacology, also known as phenotypic drug discovery, which is the historical basis of drug discovery, and reverse pharmacology, also designated target-based drug discovery. Screening methods based on phenotypic drug discovery have been used to discover new natural products mainly from terrestrial origin. Examples of the discovery of marine natural products are provided. A section on future trends provides a comprehensive overview on recent advances that will foster the pharmaceutical industry.

## 1. Introduction

The history of mankind is intimately linked to the use and exploitation of bioactive substances. The first medicinal drugs were based on plants like herbs, vines and fungi [1]. These natural products (NPs), which are essentially outcomes of the secondary metabolism of the producing organisms, were the basis for the discovery of bioactive substances. The earliest records of natural products date back to the civilizations in the Mesopotamia, Egypt, China, Greece and the Arabs [2,3]. In fact, the word “drug” is likely from Arabic origin [1].

Bioactive compounds are substances that are effective against living organisms and can be biologically produced by microorganisms, plants and animals. These substances work as chemical weapons of self-defense in very competitive environments. From a human perspective, they are important because they fulfil variable human needs. Treatment of many diseases, such as the deadly forms of cancer, and the emergence of bacterial resistance, is essentially based on active chemical principals such as antibiotics, antifungals, anticancer, antimalarial and anti-inflammatory agents. Furthermore, bioactive principles are fundamental in many industries (e.g., various enzymes [4]) and for dealing with aspects related to environmental problems such as the harmful algal blooms and fouling. These needs fostered the search for novel and/or more effective bioactive substances.

In the 2000s, the traditional NPs screening was gradually discontinued because of the frequent re-discovery of the same compounds, the technical difficulties associated with the isolation of compounds from extracts, and the incompatibility of NP extracts with high-throughput screening (HTS) campaigns. Furthermore, the structural complexity and low titer production of NPs that requires the total synthesis and derivatization are sometimes economically and synthetically challenging. As a consequence, NPs were replaced by large synthetic combinatorial libraries, mainly used in target-based screenings. However, one of the principal drawbacks of combinatorial libraries is the lack of the structural diversity and complexity given by nature to NPs. In a further step, the diversity-oriented synthesis (DOS) approach was developed to mimic NPs and the resulting compounds are currently being tested in a large number and variety of biological screens in order to determine their role as promising hits [5].

Recent advances in technology and instrumentation for the rapid identification of novel bioactive NPs and structure elucidation have opened a new era and greatly improved the NP discovery process [6]. NPs, their semi-synthetic derivatives and natural product-inspired compounds still represent one of the most important sources of chemical diversity and bioactive novel structures ever described [7]. Furthermore, and as terrestrial environments have been thoroughly searched for bioactive substances, other environments are studied for increasing the chemodiversity of NPs. Recent exploitation is now focused on the marine environment, with a very promising outcome in special using marine microorganisms from extreme environments. More than 20,000 Marine Natural Products (MNPs) have been discovered over the past 50 years [8], with about 1277 new compounds in 2016 [9] in an average of 1000 new compounds each year, after 2008 [10]. These numbers reflect the biodiversity of the life in the oceans and their potential as producers of bioactive molecules. Due to the special characteristics of the marine environment, the MNPs usually have unique chemical traits and high bioactivity [10]. Among the compounds discovered from MNPs, seven have been approved for different applications and 12 are currently in clinical trials [11]. Macroorganisms such as algae, sponges, corals and other invertebrates, as well as microorganisms have also contributed significantly towards the discovery of novel MNPs. Especially interesting are the marine invertebrates and microorganisms associated with sponges, tunicates and molluscs. The first marine-derived drug approved by the U.S. Food and Drug Administration (FDA) was cytarabine, isolated from the Caribbean sponge *Cryptotheca crypta* in 1969 for use as anticancer drug. Since then, other drugs have been approved for analgesic, anticancer, antiviral and antihypertriglyceridemia activities [12].

The screening of bioactive compounds involves a large number of analysis assays that allow assessing the potential of biological extracts or molecules. The assays can be performed at the whole animal, cell-based or molecular levels. For drug discovery, two different complementary approaches can be applied: *classical pharmacology*, also known as *phenotypic drug discovery*, which is the historical basis of drug discovery, and *reverse pharmacology* or target-based drug discovery [13]. Target-based drug discovery is based on the formulation and testing of specific molecular hypotheses [13], while in phenotypic drug discovery, extracts or compounds are assessed against, for instance, cell lines in a quantitative measurement of one or more cellular parameters [14]. Both approaches differ in their first focus. In the phenotypic approach, the function is primarily considered, and compounds are screened to find those that alter the phenotype, while the disease state and the mechanism will be identified later. In target-based approach, the gene that codes for a protein target involved in the disease is first identified and compounds are screened to find for high-affinity binding. After this, the active compounds need to be checked in tissues and whole organisms. These two strategies have advantages and disadvantages and promote very different screening assays. Complex and advanced high-content phenotypic drug screenings have been developed in which 384- or 1536-well high-content screening plates are used to screen disease-relevant cell types assembled in a biomimetic fashion [14]. Organisms like fruit fly, zebrafish or mouse are commonly used for phenotypic screenings at the whole animal level [15]. Target-based discovery benefits from the advances and evolution of science in the fields of biochemistry, structural biology, chemistry, genomics and technology.

Despite the impressive advances in technologies in the last decade, there has been a decline in the discovery of new molecular entities (NME). In the years 1996 and 2012, the U.S. Food and Drug Administration (FDA) approved, respectively, only 59 and 39 NMEs. In 2015, 45 new drugs were approved which was a higher number compared to the previous decade [16]. Furthermore, about 62% of first-in-class small molecules NMEs registered by the FDA during 1999 to 2008 had their origin in a phenotypic approach compared to 38% in a target-based drug discovery [17]. According to the data from Newman and Cragg [18], the majority of new FDA approved drugs between 1981 and 2010 were derived from NPs structures. Maybe the reasons for the failure in clinical phases are the lack of novel targets validated in preclinical stage and proper in vitro and in vivo disease models.

This review paper provides a comprehensive overview of screening methodologies commonly used and the future trends for the discovery of new bioactive compounds in the unmet medical needs in infectious and parasitic diseases, oncology and neurodegenerative diseases.

## 2. Methodologies of Screening

### 2.1. Antibacterial and Antifungal

Microbial infections have become an important clinical threat, with significant associated morbidity and mortality, which is mainly due to the development of microbial resistance to the existing antimicrobial agents. Therefore, methods for antimicrobial susceptibility testing and discovery of novel antimicrobial agents have been extensively used and continue to be developed. Some techniques were subjected to standardization by the Clinical and Laboratory Standards Institute (CLSI) and the European Committee on Antimicrobial Susceptibility Testing (EUCAST), marking the most important advances in this field [19].

In recent years, there has been a growing interest in research and development of new antimicrobial agents to combat microbial resistance. All of the most important groups of antibiotics (tetracyclines, cephalosporins, aminoglycosides and macrolides) were discovered in the 1960s and these compounds are in danger of losing their efficacy because of the increase in microbial resistance [20], due especially to the emergence of multidrug-resistant bacteria [21,22,23]. For this reason, the discovery of new antibiotics is one of the main objectives of medicine nowadays [24]. The chemical diversity associated with the marine-derived molecules is immense, from linear peptides and fatty acids to complex alkaloids, terpenes and polyketides, what makes the MNPs an amazing source for antimicrobial candidates [25].

There are some commonly used antibacterial bioassays such as disk-diffusion, well diffusion and broth or agar dilution, and other more recent methods which are not widely used to date, such as methods using flow cytometry and bioluminescence. Furthermore, one of the more commonly used in antifungal bioassays is the poisoned food technique [26]. The huge number of different assays often makes it impossible to compare results between different studies. This is further challenging by the lack of standardization in the whole procedure, which includes different media, reference strains, inoculum size and solvent used, among others. Different techniques for antibacterial and antifungal screening are summarized below.

#### 2.1.1. Agar Disk-Diffusion Method and Variations

Agar disk-diffusion is the official method used in many microbiology laboratories for antimicrobial susceptibility testing [27]. This procedure is based on the inhibition of growth of the test microorganism in agar medium plates due to the effect of the test compound. Filter paper discs are impregnated with the test compound and placed on medium surface inoculated with the test microorganism. Then, the diameters of inhibition growth zones are measured. This method accurately tests troublesome bacterial pathogens like streptococci, *Haemophilus influenzae*, *Neisseria gonorrhoeae* and *Neisseria* meningitides [28].

The advantage of this method over others is the simplicity, low cost and the ability to test great amounts of microorganisms and compounds. One of the drawbacks of this test is the inability to distinguish bactericidal and bacteriostatic effects as bacterial growth inhibition does not mean bacterial death. Furthermore, it is not appropriate for determining the minimum inhibitory concentration (MIC), because it only provides qualitative results by categorizing bacteria as susceptible, intermediate or resistant [29].

A modification of the disk-diffusion is the well diffusion method based on agar medium plates inoculated with the microorganism of interest [30,31]. In this method, the compound/extract being tested is placed into a small hole generated in the medium. If present, the antimicrobial agent diffuses into the agar medium and inhibits the growth of the microorganism.

Another modification is the gradient method (E-test), in which a strip impregnated with an increasing concentration of test compound/extract is placed on the agar surface [32]. The gradient method combines dilution and diffusion methods and is used to determine MIC and even for investigating the antimicrobial interaction between two drugs [33].

#### 2.1.2. Poisoned Food Method

In this method, the test compound/extract is incorporated into the melted agar medium at a desired final concentration. The medium is then plated into Petri dishes. When it has solidified, the fungal mycelium of interest is inoculated on the agar surface. After incubation, the diameters of fungal growth are measured and compared to the control without the compound/extract. This method is generally used to evaluate the antifungal effect and to determine the percentage inhibition of mycelial growth, minimum inhibitory concentration, and minimum fungicidal concentration [26,34].

#### 2.1.3. Thin-Layer Chromatography (TLC)-Bioautography

This technique combines thin-layer chromatography (TLC) with contact bioautography, TLC-bioautography [35,36]. This is a fast, simple, effective and inexpensive technique used for screening a large number of extracts from natural origin for bioactivity-guided fractionation [37]. This technique separates a complex mixture or an extract and, at the same time, localizes active compounds with antimicrobial activity. Some laboratories use the most sophisticated updated version of this technique with high performance liquid chromatography (HPLC), which is the method of choice for a final clean-up of active fractions to obtain pure compounds, coupled to bioautography [38].

TLC-bioautography may be used with important pathogenic strains of fungi such as *Aspergillus*, *Penicillium* and *Cladosporium*, and bacteria such as *Bacillus subtilis*, *Staphylococcus aureus* and *Escherichia coli* [39,40].

There are three different variants of this technique: agar contact method, direct bioautography and agar medium-overlay assay. In the agar contact method, the microorganism is inoculated on the agar medium plate and then the antimicrobial agent diffuses from TLC plate. After a certain period of time that can vary from minutes to hours, the TLC plate is removed and the plate with the culture incubated. The growth inhibition zones in the medium are measured [41]. The direct bioautography is the most widely used for detection of antifungal agents. It consists in dipping the TLC plate into a microbial suspension followed by incubation for 48 h. Frequently, visualization of microbial growth is performed by measurement of the conversion of tetrazolium salts by living cells [42]. The agar-overlay assay is a hybrid of the two aforementioned methods. The TLC plate is covered with agar culture medium inoculated with the microorganism of interest allowing the diffusion of tested compounds into the medium [43]. As in the previous method, staining of microorganisms can be done with tetrazolium salts.

#### 2.1.4. Dilution Method

Dilution methods are the most commonly used methods for determining MIC values against bacteria and fungi, because it is easy to estimate the concentration of the tested antimicrobial agent effective against the test microorganism.

One example of the use of this method for MNPs is the screening developed from the marine-derived fungus *Cochliobolus lunatus* in which three new eremophilane sesquiterpenes with antibacterial activity were elucidated [44].

There are two main forms of dilution method: (i) broth medium and (ii) agar dilution method. There are some approved guidelines for the dilution method to assess antimicrobial susceptibility with a uniform procedure established by the CLSI EUCAST [19].

(i) Broth dilution can be done in 96-well microtiter plates to automatize the process or in tubes with greater volumes. The antimicrobial agent is prepared in two-fold dilutions in liquid growth medium and then each tube or well is inoculated and adjusted to 0.5 McFarland scale with microbial inoculum. After microbial incubation, growth is measured. The methods usually used for this quantification are colorimetric, such as tetrazolium salts, 3-(4,5-dimethylthiazol-2-yl)-2,5-diphenyltetrazolium bromide (MTT) or resazurin dye [45].
(ii) Agar dilution consists in the incorporation of a serial two-fold dilutions of the potential antimicrobial agent into an agar medium followed by the microbial inoculation onto the agar plate’s surface. The MIC endpoint is recorded as the lowest concentration of antimicrobial agent that completely inhibits growth after incubation. Agar dilution is the recommended method for organisms such as anaerobes and *Helicobacter* species [46].

#### 2.1.5. Time-Kill Test

This method can be used to determine synergism or antagonism between bactericidal or fungicidal drugs [47]. It is the most appropriate method for determining the time- and concentration-dependent antimicrobial effect. This method has been standardized by CLSI [48]. Each drug is assayed in broth culture medium with a bacterial suspension and, after time intervals, the number of living cells is determined by the agar plate count method.

#### 2.1.6. Adenosine Triphosphate (ATP) Bioluminescence Assay

The measurement by luminescence of ATP (adenosine triphosphate), which is the chemical form of cells energy, has classically been used for cytotoxicity assays and drug discovery screening, but can be used for estimating the growth of the microbial population as well. Briefly, ATP produced by bacteria or fungi in presence of luciferase converts D-luciferin in oxyluciferin that generates light. The quantity of the emitted light is measured by a luminometer and expressed as relative light unit (RLU) which can be converted into RLU/mole of ATP. Thereby, there is a linear relationship between cell viability and luminescence measured. This technique has been used for antibacterial, antimycobacterial and antifungal testing. One of the main advantages is the rapidity; in fact, results for antimycobacterial test are obtained in only 3–5 days, in comparison with the standard dilution technique that requires 3 or 4 weeks [49].

#### 2.1.7. Flow Cytometry

Flow cytometry has been used to detect live/dead cells and some researchers use it for testing the effect of compounds/extracts in microorganisms. It is a technique that allows a rapid detection of damaged cells, and several studies have reported the effectiveness of the flow cytometer as a tool for antibacterial testing using combined staining of DNA with propidium iodide (PI) for membrane damage evaluation and carboxyfluorescein diacetate for esterase activity detection and, thus, viability [50]. The flow cytometric method allows detecting antimicrobial resistance and the estimation of the impact of the tested molecule on the viability and cell damage of the target microorganism. This technique gives reproducible results rapidly (2–6 h compared to 24–72 h for the dilution method) [51]. As this method requires specialized equipment, its widespread use is therefore unlikely.

### 2.2. Antibiofilm and Antiquorum-Sensing

#### 2.2.1. Antibiofilm Formation

Biofilms are characterized by the organized association of microbial communities attached to inorganic or biological surfaces. Additionally, the microorganisms within biofilms are embedded in a matrix enriched in polysaccharides and proteins, acting as a reservoir for the cells, providing protection against biocides and drugs and promoting drug resistance development [52,53]. Particularly, biofilm formation is a major problem within nosocomial environments due to devices microbial colonization and consequent patient infection. These devices include prosthetic heart valves, orthopedic implants, intravascular catheters, artificial hearts, left ventricular assist devices, cardiac pacemakers, vascular prostheses, cerebrospinal fluid shunts, urinary catheters, ocular prostheses and contact lenses, and intrauterine contraceptive devices [54].

The antibiofilm activity screening can be assayed through several in vitro standardized methods. For example, colorimetric-based assays using crystal violet or safranin, which are commonly used to assess extracts’ ability to disrupt biofilm formation. Especially standard protocols for both staining methods are based on identical procedures, as follows: co-cultures of extracts or compounds with indicator biofilm producing strain are performed within 96-well plates. After incubation, the wells are stained either with 0.1% crystal violet for 10–15 min [55,56] or 0.1% safranin for 1 min [57]. A washing step with sterilized water or saline solution to remove the excess staining is also needed. Regarding the resuspension of the biofilm, the approaches used are slightly different between authors: Nostro et al. [57] used 30% (*v*/*v*) acetic acid, Damiano et al. [55] 99% ethanol and Fotopoulou et al. [56] sterilized water. To determine biofilm formation, the measurement of the final suspensions absorbance is needed [55,56,57]. For example, for crystal violet staining when resuspension was carried out in water, the absorbance was read at 625 nm [56], and when resuspension was carried out in ethanol, the absorbance measured was at 495 nm [55]; for safranin staining and final resuspension in acetic acid, the absorbance was read at 492 nm [57].

Laser Confocal Microscopy can also be applied in the detection of biofilm formation. This approach relies on the incubation of a glass slide within the culture of indicator strain with extracts/compounds. The glass slide is stained with fluorescein isothiocyanate-conjugated concanavalin A for 30 min in the dark and then rinsed with sterilized water. When dried, the glass slides can be observed under a Laser Confocal Microscope [58] for the presence or absence of biofilm.

One example of an MNP is the compound isatin and derivatives that showed antibiofilm activity [59].

Using works like those here described, Papa et al. [60] reported the presence of different antibiofilm molecules in extracts obtained from marine cold-adapted bacteria.

#### 2.2.2. Antiquorum-Sensing Bioassay

Quorum sensing (QS) or cell-to-cell communication is a cell density-dependent bacterial response mediated by hormone-like compounds called autoinducers. QS-dependent regulation of gene expression controls a wide variety of prokaryotic phenotypes including biofilm formation, virulence factor expression, and motility. Quorum sensing inhibition (QSI) is considered to be a new approach of antimicrobial chemotherapy.

One example of the use of this bioassay in MNPs is the screening developed by Zhi-Ping et al. [61], in which about 200 bacterial colonies were isolated from the coral species *Pocillopora damicornis* and screened for their ability to inhibit QS using the bioreporter strain *Chromobacterium violaceum* ATCC 12472. About 15% of positives for anti-QS activity were obtained. Another MNP isolated from the marine bacterium *Rheinheimera aquimaris* showed antiquorum sensing activity through violacein assays [62].

Several methods are widely applied to evaluate the antiquorum sensing bioactivity. Some are listed and described below.

(i)  Disk-diffusion assay 

The strain *Chromobacterium violaceum* is commonly used to assess the antiquorum-sensing activity of bacterial extracts, since the presence of quorum sensing is translated into the expression of violacein which explains the purple colour of the colonies. For the disk-diffusion assays, *C. violaceum* is spread onto agar medium plates and disks containing bacterial extracts of the strains under study are plotted above the agar medium inoculated with *C. violaceum*. After incubation, antiquorum sensing activity is detected through the observation of colorless colonies of *C. violaceum* around the disks. If growth of *C. violaceum* is not observed around the discs, it is concluded that there is a bactericidal effect [63,64].

(ii) Flask incubation assay

Liquid cultures of *C. violaceum* with the bacterial extracts of the strains under study are used for the quantitative determination of violacein production. After incubation, violacein extraction is achieved by centrifugation. The resuspension of the pellet is carried out in 1 mL of dimethyl sulfoxide (DMSO) and then vortexed vigorously to dissolve the violacein completely. For the violacein quantification, absorbance is measured at 585 nm using a spectrophotometer [65].

(iii) Quorum quenching assay

The *Escherichia coli* reporter strain AI1-QQ.1 is used to identify extracts that can interfere with bacterial cell–cell communication through acyl homoserine lactone (AHL) [66]. Briefly, the *E. coli* reporter strain contains a gene that encodes a lethal protein fused to a promoter that is induced in the presence of AHL, a quorum sensing signal. Therefore, in the presence of AHL, this *E. coli* strain is unable to grow. However, when quorum sensing is disrupted, the growth of this strain will be observed. For the quorum quenching assay, an exponentially growing culture of this *E. coli* strain is incorporated into agar containing 100 μM N-(ß-ketocaproyl)-l-homoserine lactone (3-oxo-C6-HSL), 100 μg/mL ampicillin, and 30 μg/mL kanamycin, and then plated. Bacterial extracts under study are spread on the agar plates and then incubated. Quorum quenching activity is qualitatively evaluated through the presence or absence of growth of the *E. coli* reporter strain [66,67].

### 2.3. Anti-Parasitic Assays

Neglected tropical diseases (NTD) are a group of about 20 diseases that typically affect poor people in tropical countries and represent a significant health burden in large parts of the world. The available medicines to treat these diseases by no means reflect the clinical need. The complex biology of many of these parasites and their need for vectors for development and transmission makes traditional industrial-scale drug discovery programs incredibly challenging. Nevertheless, there has been a significant effort to develop new drugs to treat these diseases over the past decade and the phenotypic screens have become particularly important in NTD drug discovery because of lack of validated targets for these diseases. In this section, we have focused in three groups of organisms: kinetoplastid parasites, helminths and *Plasmodium*, because they are responsible of a large group of these diseases and have active research in drug discovery.

#### 2.3.1. Kinetoplastid Parasites

African trypanosomiasis, leishmaniasis, and Chagas disease are three neglected tropical diseases for which current treatments are inadequate and even cause toxic side effects, leading to thousands of deaths in the poorest countries. The parasite *Trypanosoma brucei*, which invades the Central Nervous System (CNS), is quite spread throughout Africa, while *Trypanosoma cruzi* is the parasite responsible for Chagas disease in Latin American countries [68]. On the other hand, Leishmaniasis is caused by different species of the genus *Leishmania* that can produce very severe infections, being fatal if untreated [69]. Millions of people are living in areas where these diseases are endemic and urgently need new drugs to help alleviate their suffering.

Drug discovery for neglected tropical diseases is carried out using both target-based and phenotypic approaches. Target-based approaches to drug discovery are extensively used in the pharmaceutical industry but there are very few fully validated drug targets for these neglected diseases [70]. Arguably, a target is only fully validated when there is a registered drug for which it can be shown that the principal mode of action is by inhibition of the target. Indeed, the mode of action of most registered drugs used for the treatment of kinetoplastid infections, such as suramin, pentamidine, melarsoprol, etc., is unknown. Even so, target-based assays are a very valuable screening tool. For instance, the drug eflornithine, included in the World Health Organization’s List of Essential Medicines [71] for the treatment of trypanosomiasis, is a “suicide inhibitor” which targets ornithine decarboxylase [72]. Therefore, there is still the need to select new targets among those involved in highly essential biological pathways of kinetoplastid.

One of the targets used is the enzyme 3′,5′-cyclic nucleotide phosphodiesterase (PDE) type B1 from the parasite *Trypanosoma brucei* (TbrPDEB1). PDE is an essential protein for the proliferation of the parasite [73]. The enzyme regulates cyclic nucleotide levels and induces the cessation of the parasite cell division, with consequent lysis and control of the infection in a mouse model [74]. Additionally, the human PDE type 4D (hPDE4D) activity should be assayed in parallel for the compounds screened to avoid off-target effects. Another target used for *T. brucei* is methionyl-tRNA synthetase (MetRS) [75,76] which plays a crucial role in protein synthesis and has been validated in a mouse model [77]. Furthermore, this enzyme is highly conserved among kinetoplastid parasites. The effect of the compounds screened in protein synthesis of human cells has to be assayed in parallel. Other examples of target-based assays are the inhibitors of sterol 14-α-demethylase which have been shown to be active against Chagas disease [78] and *Leishmania* casein kinase 1.2 (LmCK1.2), an exoprotein kinase that has recently been shown to be essential for intracellular parasite survival and infectivity [79].

However, the most widely used methods for drug discovery against kinetoplastid are phenotypic [80]. The kinetoplastid parasites have complex life cycles. They may be free-living or parasitic, with several stages that occur between the insect vector and vertebrate host. The drug discovery screening can be performed in any of these stages with varying results. Such assays include the use of the free-living promastigote parasite (from the insect stage) or amastigote parasites (from mammalian stage) from an axenic culture or in an intracellular stage (the more physiologically relevant). The most frequently used assays are developed using the mammalian stage of the parasites, such as the intracellular amastigote form of *T. cruzi*, the bloodstream form of *T. brucei*, and both axenic and intracellular amastigote forms of *Leishmania donovani*. The screening methods range from tests with less performance, such as microscopic observation, to high performance assays, such as HTS in 384-well format [81]. The models used for drug discovery screening can be parasites genetically engineered to express reporter gene such as *E. coli* β-galactosidase gene, *lacZ* [82] or parasites that have a stable expression of luciferase activity in mammalian host cells [83]. Additionally, dyes indicators of viability such as resazurin are used for testing drug susceptibilities of the parasites [84]. The recent advances in automated microscopy have the capacity to increase throughput by replacing laborious manual microscopic observation for high-content imaging and this technique is being successfully used as in vitro whole-organism screens against live kinetoplastid parasites [85].

Most drug discovery screenings for anti-kinetoplastid drugs are performed with synthetic compound libraries to search for active compounds [86] but such synthetic libraries are often limited in structural diversity and novelty. Natural products offer an alternative source of highly underexplored chemical entities with privileged bioactive molecules that could be used as templates for the synthesis of novel drugs in the treatment of the tropical diseases [81]. Furthermore, there are also numerous natural products with promising anti-kinetoplastid activities which await being developed as drugs [87]. There are some examples of marine origin compounds effective in drug discovery screening for anti-kinetoplastid drugs such as Iotrochamides A–B, from the Australian marine sponge *Iotrochota* sp. [88], Convolutamines I–J from the bryozoan *Amathia tortusa* [89] and others isolated from the Indo-Pacific marine sponge *Cacospongia mycofijiensis* [90].

#### 2.3.2. Helminths

Helminths are parasitic worms. They are one of the most common infectious agents of humans in developing countries. The helminths are responsible for infections such as filariasis, schistosomiasis and ascariasis. Little is known about the unique biochemical metabolism of parasitic worms and the mechanisms by which worms evade human host defenses and establish chronic infections. Although there are millions of people infected by these parasites, there are limited tools for controlling worm infections; of the 1556 new chemical entities marketed between 1975 and 2004, only four drugs (albendazole, oxamniquine, praziquantel, and ivermectin) were developed to treat helminthiases [91]. Among these four drugs only ivermectin is a natural product, derived from the bacterium *Streptomyces avermitilis*. In the last decade, some screening campaigns has been developed looking for anthelminths from marine natural products and, for instance, a potent nematocidal activity has been described for new thiocarbamate thiocyanates, thiocyanatins E1 and E2 from a southern Australian marine sponge, *Oceanapia* sp. [92].

To date, the majority of the approved anthelmintics have been derived from veterinary medicine and discovered through in vivo animal model testing. However, recent approaches have turned towards screening in adult or larval stages in vitro, even though target-based screening, with a focus on thioredoxin glutathione reductase inhibition, has been successfully applied in the identification of oxadiazoles as new lead compounds for schistosomiasis [93].

Some species of helminths are difficult to grow in the laboratory so other free-living nonparasitic species are used as models for these organisms. One example is *Caenorhabditis elegans*, which is used extensively as a model to identify drug targets and potential novel anthelmintics because it can be readily cultured in vitro [94]. The larvae of helminths are normally available in greater numbers than adults and are small enough for microplate assays. These characteristics, together with the implemented high-content imaging techniques, have made it possible to use large compound collections to be screened against helminths, revitalizing the drug discovery pipeline [95]. Both light-field and fluorescent-based assays have been performed for testing the viability of the parasites after drug treatment. With the aim of reducing the subjectivity of the images, some vital dyes enable quick identification of dead/live parasites, such as methylene blue and trypan blue. Additionally, indicators of metabolic activity such as the MTT (3-(4,5-Dimethylthiazol-2-yl)-2,5-diphenyltetra-zolium bromide) [96], Alarm blue [97] and acid phosphatase activity have been widely used to assess viability of some helminth species.

#### 2.3.3. Malaria

Malaria is a mosquito-borne infectious disease affecting humans and other animals caused by parasitic protozoans belonging to the *Plasmodium* genus. Malaria is widespread in tropical and subtropical regions, including America, Asia and Africa. According to the “World Malaria Report 2016” of the World Health Organization, there were 212 million new cases of malaria worldwide in 2015. Most deaths from malaria are caused by *Plasmodium falciparum*, one of the five species of human infectious malaria parasites. The increasing resistance of *P. falciparum* to the available drugs and new efforts to eradicate malaria drive the need to develop new and effective antimalarial drugs [98]. Traditionally, plants have been excellent sources of antimalarial compounds. In fact, artemisinin, a natural product isolated from the *Artemisia annua*, used in Traditional Chinese medicine, is the drug recommended by the World Health Organization to be used in combination therapies as a first-line treatment of malaria. The combination therapy decreases drug resistance because the combination of antimalarial drugs with independent modes of action can impede the development of resistance to each individual component of the combination [99], increasing the probability of success.

New antimalarial drugs should be effective against several of the developmental stages of the parasite. Following the blood meal of an infected *Anopheles* mosquito, the *Plasmodium* sporozoites travel through the bloodstream of the mammalian host. When they reach the liver, they invade the hepatocytes and transform into an exoerythrocytic stage. At this stage, the parasites can leave the liver and re-enter in the bloodstream in an asexual blood stage, where they can cause red blood cells destruction and the characteristic symptoms associated with malaria: anemia, fever, and chills. A small percentage of these asexual blood stage parasites will then differentiate into sexual erythrocytic-stage parasites as female and male gametocytes. The transmission of the sexual blood stage back to the mosquito vector during a subsequent blood meal completes the *Plasmodium* life cycle [100].

Several drugs with antimalarial activity are under development. The Medicines for Malaria Venture Malaria Box is a collection of over 400 compounds representing families of structures identified in phenotypic screens of pharmaceutical and academic libraries [101]. This library was originated for the screening against the asexual-stage of *Plasmodium falciparum* by two pharmaceutical companies, GlaxoSmithKline and Novartis, and two academic centers, St. Jude (Memphis) and Eskitis (Australia) and now it is available for malaria researchers to request it to perform tests on their malarial screens, with the only condition that information obtained be deposited in the public domain. All the results deposited were summarized by Van Voorhis et al. [102], suggesting a potential mechanism of action for over 130 compounds against malaria and identifying the best for further malaria drug development research. Additionally, due to the low structural diversity within the antimalarial drugs currently available in the clinic and the increasing number of cases of resistance, NPs are important sources for new chemical scaffolds in antimalarial agents [103].

The phenotypic screening assays are generally developed in asexual erythrocytic-stage parasites with different readouts for the inhibition of parasite growth such as lactate dehydrogenase (LDH) release, one of the most commonly used due to its robustness and specificity [104], MitoTracker or Sybr Green dye incorporation, hypoxanthine incorporation or 4’,6-diamidino-2-phenylindole (DAPI) imaging assay or also using a transgenic *P. falciparum* line that stably express cytoplasmic firefly luciferase that was used to develop a cell-based luminescent method for testing antimalarial drugs which can be adapted to HTS format [105].

Alternative, other stages of *Plasmodium*, such as liver stage, are attractive targets for the development of antimalarial drugs and it is an opportunity to interrupt the life cycle of the parasite at a critical early stage. Both *P. falciparum* and *P. vivax* have been established in a microscale human liver platform composed of cryopreserved human primary hepatocytes surrounded by supportive stromal cells. [106]. This system allows a more complete study of the effect of the drugs in the *Plasmodium* liver stage, in the release of merozoites and infection of erythrocytes, using image automation methods. Other approach, in this context, is the use of pluripotent stem cells (iPSC) differentiated to iPSC-derived hepatocyte-like cells (iHLCs) as in vitro model of liver-stage malaria of *P. berghei*, *P. yoelii*, *P. falciparum*, and *P. vivax* species [107].

Additionally, the intervention over gametocyte development, which directly blocks the parasite transmission, has been used as drug screening method. One of the novel methods described for the identification of anti-gametocyte compounds is the measurement of the parasite lactate dehydrogenase activity of gametocytes of *P. falciparum* in 96-well plates as an indicator of gametocyte viability [108].

At molecular level, there are some validated drug targets to fight against malaria. These include dihydrofolate reductase and dihydropteroate synthase [109], the atovaquone targeting the mitochondrial bc1 complex [110] and the enzyme involved in pyrimidine biosynthesis, dihydroorotate dehydrogenase [111].

There are some examples of marine origin compounds effective in drug discovery screening for antimalarial drugs such as thiaplakortones A–D from the Australian marine sponge *Plakortis lita* [112].

More validated targets and better HTS assays could help to develop new drugs for treating these neglected diseases faster.

### 2.4. Anticancer

Cancer is a broad term for several diseases affecting any part of the body such as lungs, liver, stomach and breast. Cancer is defined as a serious disease that is caused when cells in the body grow in a way that is uncontrolled and not normal, killing normal cells and often causing death. The modification from normal cells into tumour cells is the initial multistage process that leads to a continual unregulated proliferation of the cells. The tumour cells are able to grow abnormally and without boundaries, spreading easily into neighbour tissues and organs [113].

Cancer was responsible for 8.8 million deaths in 2015 worldwide, being the leading cause of death in the world [113]. Consequently, new anticancer agents with more effectiveness and less toxicity are urgently demanded. Microbial NPs have been explored as an answer to these pharmaceutical needs with promising results, in addition to the synthetic compounds libraries used for cancer drug discovery.

Drug discovery for cancer is carried out using both target-based and phenotypic approaches. Target-based approaches to drug discovery are extensively used in the pharmaceutical industry but there are few fully validated drug targets in some types of cancers that are dependent on the tumour microenvironment, such as pancreatic cancer [114]. In others, such as breast cancer, estrogen receptor is one of the targets screened or, in most types, the blocking of microtubule proteins such as tubulin is a pursued target for interfering in the mitosis of the cells [62]. Due the lack of avowed target in some cancers and the advances in cell culture techniques, the most widely used approaches in cancer research are phenotypic.

In the phenotypic approach, the first steps of the research for anticancer leads rely on the in vitro assessment of anticancer activity. Established cell lines of each type of cancer are commonly used to carry out by HTS assays, while normal cell lines are used as control. In many types of cancer, such as pancreatic cancer, the subpopulation of cancer stem cell (CSC) is highly enriched. CSCs are resistant to current chemotherapeutic drugs and therefore promote tumour recurrence [115]. CSCs, also called tumour-initiating cells, share many characteristics with normal stem cells, such as, asymmetric cell division, in which each CSC generates one daughter cell with self-renewal capacity and another cell destined to differentiate. The self-renewal capacity helps to maintain the number of CSCs within the tumour. CSCs also exhibit unique features, such as their metastasis ability and the ability to remain in a quiescent state, which protect them from the chemotherapeutic drugs developed to target actively dividing cells. Nowadays, the use of CSCs in anticancer screening is becoming the most relevant phenotypic approach.

Therefore, there are different strategies for drug discovery in cancer research using established cancer cell lines in 2D cultures or CSCs-based approaches using 3D cultures (spheroids) [116] and high-content imaging techniques, which are explained in more depth in Section 3. Interestingly, a pilot screen to validate a 3D model of breast cancer metastasis for HTS using a purified library of marine metabolites has been reported, with excellent results [117]. In this study, four hits, isonaamidine B, papuamine, mycalolide E, and jaspamide were confirmed.

High-throughput screening for anticancer compounds are usually developed in 2D cultures, where cancer cell lines are exposed to the extracts or compound(s) and then are tested for cell proliferation and cytotoxicity assays [118,119], like the ones described on the following sections.

#### 2.4.1. Stained Viable Cells Assay

Adherent cells detach from cell culture plates during cell death and this characteristic can be used for the indirect quantification of cell death and to determine differences in proliferation upon stimulation with death-inducing agents. A simple method to detect maintained adherence of cells is the staining of attached cells with crystal violet, neutral red and sulforhodamine B dyes.

Crystal violet binds to proteins and DNA. When cells that undergo cell death are detached from cell plate, the amount of crystal violet staining is reduced [120].

The neutral red uptake (NRU) assay provides a quantitative estimation of the number of viable cells in a culture. The ability of viable cells to incorporate and bind the supravital dye neutral red in the lysosomes is measured by colorimetry [121].

The bright pink aminoxanthene dye sulforhodamine B (SRB) is able to bind to proteinaceous components of cells that were previously fixed with trichloroacetic acid (TCA). Despite not being able to discriminate viable from dead cells, SRB assay is widely used for cell density determination, being extensively used for cytotoxicity screenings. Some studies have assessed the SRB assay ability to evaluate cytotoxic effects of compounds by comparing SRB assay results with those obtained with metabolic assays such as MTT test [122,123,124]. These comparative studies showed a high correlation between results of both assays, with the difference that IC_50_ values of compounds under test were slightly higher with SRB assay [122,123,124]. Additionally, SRB assay is ideal to be carried out in multi-well plates [125].

In all the cases, the dye is extracted in each well and the absorbance is read using a spectrophotometer. All these procedures are cheaper than other cytotoxicity tests such as tetrazolium salts.

#### 2.4.2. Dye Exclusion Assay

The number of viable cells after exposure to a compound under test can be carried out by dye exclusion methods. These methods rely on the integrity of the cell membrane, which is responsible for the exclusion of certain dyes like trypan blue, eosin or propidium iodide in live cells. On the other hand, dead cells with damaged cell membranes are able to uptake the dye, being coloured.

For example, on the trypan blue exclusion assay, pellets of the cell lines exposed to the compound under test are resuspended in phosphate buffered saline (PBS), diluted in a solution of 0.4% trypan blue (1:1) and incubated for 3 min at room temperature. Observation under a light microscope allows the count of viable and dead cells with the help of haemocytometer or with more sophisticated automatic cell counters. A simple calculation of the percentage of viable cells through the formula *viable cells (%) = total number of viable cells per mL/total number of cells per mL* will unveil the effect of the compound [126].

#### 2.4.3. Methods Based on Metabolic Activity

There are three widely methods to assay cytotoxicity based on metabolic activity of cell lines: tretrazolium reduction, resazurine and ATP content assay.

(i)  Tetrazolium reduction assay

This technique needs an incubation of the tetrazolium compounds with cell cultures, in which the viable cells are able to convert these compounds into brightly coloured products that can be colorimetrically detected with a conventional microplate reader [127,128]. In this type of assay, the most commonly used compounds of the tetrazolium family are MTT, 5-[3-(carboxymethoxy)-phenyl]-3-(4,5-dimethyl-2-thiazolyl)-2-(4-sulfophenyl)-2H-tetrazolium inner salt (MTS), sodium 2,3-bis(2-methoxy-4-nitro-5-sulfophenyl)-5-[(phenylamino)-carbonyl]-2H-tetrazolium inner salt (XTT) and sodium 5-(2,4-disulfophenyl)-2-(4-iodophenyl)-3-(4-nitrophenyl)-2H-tetrazolium inner salt (WTS-1). Particularities of each dye assay are specified by Riss et al., Goodwin et al. and Boivin et al. [128,129,130]. Additionally, to perform these assays, there are several standardized ready-to-use kits that are commercialized.

(ii) Resazurin assay

Resazurin is a cell health non-fluorescent indicator, but upon entering living cells, resazurin is reduced to resorufin, a highly fluorescent red compound. Changes in viability can be easily detected using either an absorbance- or fluorescence-based plate reader. Resazurin is the active ingredient of alamarBlue^TM^ (Invitrogen, Carlsbad, CA, USA) ready-to-use reagent [131].

(iii) ATP content assay

The adenosine triphosphate (ATP) has a key role in cellular biological processes, being the main carrier of energy in cells. Additionally, ATP concentration in all living cells is similarly well correlated with cell biomass [132]. When cellular membrane loses its integrity, the ability to produce ATP is also lost and the remaining ATP is quickly consumed by endogenous ATPases. Therefore, ATP assay has been widely used as a marker to detect viable cells and is also the most frequently used method to assess cell viability through high-throughput screening platforms [128].

For the ATP assay, the use of an ATP detection kit is crucial. This kit includes a detergent for cell lyses, ATPases inhibitors to prevent depletion of the ATP released from lysed cells, luciferin as substrate and luciferase that is responsible for the enzymatic reaction that generates bioluminescence. Briefly, the ATP assay consists in adding the ATP detection reagent to the cell culture exposed to the compounds under test and, after an incubation of 10 min, the absorbance is recorded at 570 nm [128]. One of the most sensitive reagents is CellTiter-Glo^TM^ (Promega, Madison, WI, USA), which is a luminescence reagent used to determine the number of viable cells in culture based on quantitation of the ATP present in cell culture by a luminometer.

#### 2.4.4. Protease Viability Marker Assay

A wide range of enzymes are present in mammalian cells, such as lipases, nucleases and proteases. Proteases are usually compartmentalized inside the cell, according to their specific function, and are active while the cell is viable [128]. Therefore, there are two different approaches to use the proteases as a biomarker for cell viability:

(i) Measuring the viability of the cells, by a protease assay, using a cell permeable fluorogenic protease substrate that will penetrate the cell and serve as a substrate for the proteases inside the cell, marking then the viable cells.
(ii) Measuring the cytotoxicity through the protease activity, using substrate that reacts with proteases released into the external media, thus assessing the protease activity of compromised cells.

Glycyl-phenylalanyl-amino-fluorocoumerin (GF-AFC) is one of the substrates most often used for marking viable cells [133] (i). This method is more sensitive, less time consuming and not as toxic as, for example, tetrazolium reduction assays. Therefore, it is becoming more common. Additionally, the fact that this assay can be used in combination with other assays is a big advantage over other methods. GC-AFC enters viable cells where it is transformed by cytoplasmic aminopeptidase and aminofluorocoumarin (AFC) is released, generating a fluorescent signal, proportional to the number of viable cells. GF-AFC is usually prepared in a neutral buffer and the generated fluorescence, which corresponds to the viability, can be measure in a fluorometer with 380–400 nm excitation source/505 nm emission filter set [134].

For measuring the cytotoxic effects (ii), using the protease activity, for example, *bis*-Ala-Ala-Phe-R110 (AAF-R110) can be used as substrate. This substrate is not able to enter the cells, reacting only with proteases present in the external media, which have been released into the media by dead cells. As in the GF-AFC assay, the substrate is cleaved by proteases which leads to a fluorescent signal (rhodamine 110–R110). This fluorescence can be measured by a fluorometer at 485 nm excitation and 520–530 nm emission filters [135].

As referred to above, the GF-ACF assay can be used in multiplex with other assays. In fact, cell cytotoxicity can be measured, through the AAF-R110 assay, together with the viability AFC fluorescence assay to have a more complete range of results [133,134].

#### 2.4.5. Clonogenic Cell Survival Assay

Clonogenic cell survival assay was initially developed for screening the effects of radiation in mammalian cells, but nowadays it is used as a tool for studying the effect of compounds on tumour cells. A cell is considered clonogenic if it maintains its ability to proliferate indefinitely and to form a clone or colony. If a cell is able to form a visible colony to the naked eye that means that it has maintained its capacity to proliferate [136]. The loss of reproductive integrity can be related to the antitumor capacity of compounds by a dose-survival curve [137].

This assay consists in plating a known number of cells after treatment, allowing them to settle and grow, staining them, and counting the cells that survived and started a colony. This is measured in plating efficiency (the percentage of cells seeded into a plate that formed a colony/clone). Colonies that appear to have damage in the nucleus or that are only formed of one or two cells should be considered dead, as they are not able to reproduce indefinitely [137,138].

#### 2.4.6. DNA Synthesis Cell Proliferation Assay

The absence of cell proliferation can reflect the toxic effect of a compound in a cell population and, therefore, assessing it can be useful when testing new anticancer molecules. One of the most reliable and accurate proliferation assays is measurement of DNA synthesized in the presence of a label. Traditional cell proliferation assays involve thymidine analogues, by incubating cells with ^3^H-thymidine. Proliferating cells incorporate the radioactive label into their newly synthesized DNA, which can be measured using a scintillation counter, comparing the division of treated cells with the control [139,140]. Downsides of this method are the excessive length of the experiment and the impossibility for the method to be performed in vivo*,* only in vitro or ex vivo*,* and that no further studies can be done with the cells. Furthermore, nowadays, the use of radioactive materials is avoided and another protocol, using 5-bromo-2′-deoxyuridine (BrdU), also a thymidine analogue, is usually performed. BrdU is also incorporated into newly synthetized DNA and is detected by BrdU-specific antibody, sometimes followed by a secondary antibody as a reporter, before it can be measured as a colorimetric, chemiluminescent or fluorescent reporter signal. This assay is suitable for immunohistochemistry, immunocytochemistry, in-cell ELISAs, flow cytometry analysis and HTS. Besides avoiding the use of radioactivity, the advantages of this method are the possibility of recovering the DNA through the antibody with posterior use not only in vitro but also in vivo*.* The use in live animals can be done by the addition of BrdU into the water or upon injection, and, since it is incorporated in the DNA, it can persist for a few months. Moreover, if other stains are used it is possible to select a specific type of cell to be studied. Disadvantages of these two assays are their endpoint assays nature and their incapacity to identify cells that divided more than once [140].

Other commercial reagents are usually applied for quantifying DNA, such as PicoGreen^TM^ (Molecular Probes, Eugene, OR, USA), which quantifies double-stranded DNA. This assay is very sensitive and can be measured using a standard fluorometer or a microplate reader.

In addition to these classic methods, the tendency in drug discovery in oncology is the use of more complex cell-based models such as 3D cultures and microfluidic systems, which are addressed in Section 3, Future Trends.

Numerous studies have reported MNPs showing anticancer activities, which were reviewed by Chen et al. [141]. In fact, several MNPs for cancer treatment were also tested in human clinical trials, such as soblidotin, girolline, aplidine, discodermolide, sarcodictyin, thiocoraline, ascididemnin, manoalide, or even didemnin, which was the first MNP analysed in human clinical trials [142].

### 2.5. Neuroprotectors

Neurodegenerative diseases (NDs) comprise a collection of disorders with chronic and progressive loss of neural function, manifesting a large variety of clinical symptoms depending on the population of neurons affected. The etiology of neurodegeneration is quite heterogeneous [143]. The most common NDs are Alzheimer’s disease (AD) and Parkinson’s disease (PD), affecting millions of people worldwide. One of the main problems to find a cure is the incomplete understanding of the molecular mechanisms underlying neurodegeneration which results in a general lack of well-validated targets. Consequently, the main goal is to discover the complete molecular mechanisms of the diseases and, therefore, to discover new targets. Nowadays, drug discovery for NDs is focused on identifying symptomatic or disease-modifying molecules which, even if they do not cure the disease, at least delay or halt disease progression. In fact, few treatments are available for NDs and most of them only control the symptoms in the early phases of the disease. Examples are donepezil, which controls dementia for a short period of time in AD patients [144], and levodopa for PD patients. Additionally, another obstacle in translating drug discovery to clinical treatment for NDs is the need for the active compounds to cross the blood-brain barrier (BBB).

Target-directed and phenotypic drug discovery screens have been broadly used for drug discovery but in the case of NDs, modulating a single gene target may not always be the best disease model and the active molecules barely have a therapeutic impact, especially for NDs with complex mechanisms [145]. Blocking a single pharmaceutical target with a high-affinity drug is, in theory, the best approach to identifying an effective drug candidate, because it minimizes undesirable side effects. However, this is not always true, since the drug targets are likely to have additional biological functions required for normal functions, thereby leading to toxicities. Another disadvantage is the possibility of occurrence of false positives in protein-target screenings with, for instance, plant-derived natural products due to the abundance of hydrophobic small molecules such as polyphenolics, as they can bind nonspecifically to some protein targets because of their “sticky” nature. Anyway, both approaches are used for NDs drug discovery.

In AD, target-based approaches have limited success since the majority of the screenings are focused on the development of high-affinity novel compounds that can potentially inhibit known AD targets related to amyloid cascade hypothesis [146]. The central player in this approach is amyloid beta (Aβ) regarding its formation, fibrillization, aggregation and the formation of the senile plaques as well as the factors that control and contribute to these processes, such as secretases [147]. Twenty-five years after the enunciation of amyloid cascade hypothesis [148], no drugs working efficiently against AD have been discovered. Another key factor of AD is tau hyperphosphorylation [149] and some screenings aimed at preventing this phosphorylation have been unsuccessful. Despite the time elapsed since the discovery and the small degree of success, several “pharmas” continue with these approaches. Another target used for AD is acetylcholinesterase (AchE) [150]. Decline in cholinergic activity in the brain of Alzheimer patients is associated with cognitive decline and several of the AD drugs currently on the market are targeting this dysfunction (galantamine, rivastigmine, etc.). One marine-derived compound, 4-acetoxy-plakinamine B, isolated from the Thai sponge *Corticium* sp., was reported to inhibit AChE [151].

Similarly, PD is characterized by a key pathology of intracellular Lewy bodies composed of α-synuclein [152] and the loss of dopaminergic function [153]. Although the function of α-synuclein has not been fully elucidated, several studies have suggested that α-synuclein accumulation induces dysfunction of neurons and α-synuclein has been the target of some high-throughput screenings, along with the search for dopamine agonist.

On the other hand, the previous successes of phenotypic screens in Central Nervous System (CNS) diseases were serendipitous findings or discovered through animal tests, with limited throughput. Nowadays, the accumulated knowledge on the biological pathways involved in the neurodegeneration process shows that multiple neurodegenerative diseases have many features in common, including mitochondrial dysfunction, abnormalities in protein degradation pathways, axonal transport defects, and the ultimate induction of cell death pathways. This knowledge, together with the great advance on screening technologies, has contributed to develop phenotypic screenings with higher throughput in cellular, organotypic or small animal models (e.g., *Caenorhabditis elegans*, *Drosophila*, and zebrafish) [154,155]. These screens provide the opportunity to identify molecules that can modulate the biological pathways related to disease progression in ND.

There are three major phenotypic screening strategies: Stress reduction; Neuroprotection; and Regeneration.

#### 2.5.1. Stress Reduction

The aim of this approach is to eliminate the stresses or toxins that cause neurodegeneration. The hypothesis is that by reducing the stress/toxin levels in the cells or in the surrounding environment, the affected neurons will be able to self-recover and recuperate functions. Therefore, reduction of stress levels is the primary endpoint. An example of this approach is the screening for drugs for Huntington’s disease (HD). HD is caused by autosomal dominant mutation of the Huntington gene (*Htt*), characterized by the expansion of CAG triplet repeats. The mutant Htt is prone to aggregation, and generates intranuclear inclusions in striatal medium spiny neurons, causing cell death [156]. Based on this phenotype, a high-throughput assay was designed to quantify intracellular Htt aggregates in PC12 cells expressing mutant Htt [157]. In the case of AD, the pathological hallmarks are amyloid plaques composed of Aβ peptides and intracellular neurofibrillary tangles composed of hyperphosphorylated stabilizing microtubules tau proteins, so phenotypic screens have been done to seek for compounds that reduce Aβ or tau levels, using an established cell line that secretes Aβ [158] or overexpresses tau [159]. The MNPs glycine betaine derived from diverse marine organisms such as the red alga *Ceratodictyon spongiosum*, and the mollusca *Patella vulgate*, was reported for the reduction of tau hyperphosphorylation and Aβ deposition by inhibiting BACE1 [160]. In the case of PD, which is characterized by progressive degeneration of dopaminergic neurons in the *substantia nigra*, the loss of dopaminergic neurons results in diminished dopaminergic stimulation, causing motor dysfunction. Therefore, agents that improve degeneration of neurons are actively sought to modify disease progression and provide clinical benefit. In the case of the marine-derived compound, 11-dehydrosinulariolide, isolated from the cultured soft coral *Sinularia flexibilis*, it was reported the protection over an in vitro PD model in human neuroblastoma cells against 6-hydroxydopamine-induced cytotoxicity [161].

Other than typical pathological hallmarks, phenotypic screening are useful for searching compounds with antioxidative [162] and anti-neuroinflammatory properties [163]. Antioxidative properties are easily assayed in HTS formats measuring the reactive oxygen species (ROS) formed in cell-based assays [164] or in chemical assays such as the α, α-diphenyl-β-picrylhydrazyl (DPPH) free radical scavenging method [165] and oxygen radical absorbance capacity (ORAC) method [166]. One marine-derived antioxidant, NP7, has been reported to be a potent free radical scavenging agent and to protect neuronal and glial cultures from H_2_O_2_-induced cell death [167].

On the other hand, neuroinflammation is characterized by glia cell activation, peripheral lymphocyte infiltration and increased proinflammatory cytokine levels [168]. Using a microglia-like cell line BV-2 or similar, compounds with anti-inflammatory properties can be screened against lipopolysaccharide (LPS)–induced nitric oxide production, tumour necrosis factor–α and interleukin-1β [163].

#### 2.5.2. Neuroprotection

Neuroprotection is designed to look for molecules that directly protect the neurons against disease-relevant insults. Protection of cell death is the primary endpoint for these screens. This approach works only in dysfunctional neurons that can still be rescued from disease before neuronal damage becomes irreversible. Therefore, neuroprotection is another important strategy for preserving the remaining functional neurons in a maximal way and slowing down the disease progression. An example of drug discover for AD is screening compounds that avoid the in vitro neuron damage triggered by extracellular Aβ [169]. In the case of PD, cell culture is treated with 1-methyl-4-phenylpyridinium (MPP+), which can specifically induce dopaminergic neuron death [170], or with rotenone which triggers mitochondrial dysfunction [171]. One marine-derived compound, Secalonic acid A, present in marine fungi such as *Aspergillus ochraceus* and *Paecilomyces* sp., protects against MPP+-induced neurotoxicity in cell line models [172]. Other properties of the compounds tested are assayed in throughput screens using cell-imaging techniques such as high-content screening (HCS) that has been widely applied to monitor neuronal-specific changes, such as neurite length, branching, or synapse formation [173].

Cell-based assays may not be sufficient to represent all the aspects of the disease, so organotypic culture, organoids and animal models are used as secondary assays to confirm hits from primary assays. These models offer a much more physiological environment for studying the interaction of different cell types, tissues and systemic factors. For example, zebrafish, a small vertebrate, is used as a model for Parkinson’s disease when treated with 1-methyl-4-phenylpyridinium (MPP+) [174].

#### 2.5.3. Regeneration

Regeneration seeks compounds that promote neurogenesis through differentiation of neural stem cells into neural network [175]. Neural progenitor cells (NPCs) from rodent brains provide an available source of cells for neurogenesis screens [176]. In the last few years, the advance of induced pluripotent stem cells (iPSC) technology has made it possible to obtain NPC cell lines from iPSC reprogrammed from healthy or patient cells such as fibroblasts, opening a new field of research [177].

In conclusion, because most NDs must be considered as multifactorial diseases, the therapies will gain in potency if combined with multitarget treatments. Therefore, screening strategies should combine phenotypic and target-based approaches to increase the likelihood of success. Furthermore, the phenotypic screening strategies could potentially reveal new drug pathway relationships and specific molecular targets that may lead to new disease targets in NDs which could be used in future combined screening projects.

Table 1 summarizes, for the different screening methods, the respective advantages and disadvantages.

## 3. Future Trends

The periodic emergence of infections and antimicrobial resistance makes new disease-relevant cell-based phenotypic assay methodologies necessary to enhance the knowledge of the dynamics of host–pathogen interactions in their natural environment and the development of new therapies. In addition, even though there have been many notable drug discovery and development achievements in recent years, several disease areas such as neurodegeneration and aggressive cancers remain largely intractable. This failure can be attributed, in part, to our limited understanding of the targets of diseases and to a lack of robust disease-relevant screening methods that mimic the key pathophysiological features of the human disease. Therefore, current efforts should be directed towards the development of new models, new assay formats and innovative screening technologies that better summarize in vivo physiology [178].

While all the early drugs were discovered by phenotypic screening, the past three decades have given rise to new technologies for making large chemical libraries (combinatorial chemistry) and high-throughput screening (robotics) that have since dominated the pharmaceutical industry. Future trends in drug discovery and development includes systematic computational analyses mimicking ecosystems of the pathogens and patient-derived cell cultures, induced pluripotent stem cell (iPSC) technology, three-dimensional co-culture, organotypic systems, advances in cell imaging, microfluidics, nanotechnologies and gene editing technologies.

Among the computational systems used for drug discovery, dynamic combinatorial chemistry (DCC) is a powerful tool for hit identification and optimization for the discovery of binders to DNA, RNA and protein targets, including biological macromolecular targets as receptors [179]. The DCC technique involves the generation of a library of compounds by reversible reaction of different building blocks. The main advantage of DCC is that several potential ligands for a protein can be screened simultaneously, avoiding the individual synthesis of every compound. This emerging technology has been used for bacterial targets [180] and cancer targets such as VEGFR [181]. Another strategy is the use of drug repositioning, which is a process of discovering a new therapeutic use for existing drugs, allowing the prediction of novel targets and therapeutic indications [182]. The virtual access to a large number of compounds and data relating to the target disease and informatics-based approaches can complement and further facilitate drug repositioning efforts more systematically than the experimental approaches. The key objective of the computational approach is to identify novel drug–disease connections using several computational methods, such as the transcriptomic or genome wide association studies (GWAS). In the case of transcriptomic approach, gene expression profiles serve as a regulatory “signature” consisting of genes either up- or down-regulated in the disease state compared with unaffected controls. This approach is based on the “connectivity map” project, which first built a database of gene expression profiles associated with a number of reference drugs [183]. The in silico comparison of these disease signatures with drug-specific signatures can enable the prediction of new indications for a particular drug. Secondly, GWAS reveal new information regarding the association of specific genomic variations with complex trait human diseases, determining a subset of genes considered to be “drug targets” based on the druggability of each gene product and predicting new targets [184].

A way to develop more physiological infectious disease models is to mimic as closely as possible the in vivo ecosystems of the pathogens when infecting the host, to identify drugs that might boost the host defense mechanisms or act directly in the pathogenic stage of the microorganism. Many pathogens have been studied in animal models such as *Drosophila melanogaster*, *Caenorhabditis elegans* or *Danio rerio* (zebrafish) but unfortunately, they do not mimic properly the human response. There are emergent models using synthetic microtissues that overcome the drawbacks of these animal models, such as the human gut-on-a-chip [185].

Patient-derived cell cultures offer a more clinically relevant model for testing novel gene and cell-based therapies. These models are decidedly valuable in cancer research, where highly selective drugs targeted at genetically defined clinical subtypes are needed to support a more patient-centric approach to drug development [186]. Potential drugs have been tested against patient-derived primary cancer subtypes for various cancers, with promising results in glioblastoma [187] and leukemia [188]. The problem is that the in vitro cell culture conditions modify cells over time, losing the expression of markers or enriching specific cell populations. One solution for that is to use primary ex vivo cell cultures, which is difficult for an extensive research, or to use living organoid biobanks, which consist of a rapidly growing collection of organoids from patients with various forms of cancer that closely recapitulate several properties of the original tumour [189].

However, the availability of the relevant tissue and the scaling-up of the cultures for HTS are limiting factors, even most notably in the field of neurodegenerative disease research. A solution to this handicap came with iPSC technology, where the expression “disease in a dish” was coined [190]. These cells represent normal primary cells with a fairly stable genotype and with the capacity for self-renewal, facilitating their expansion for drug screening [191]. Another advantage of iPSC is that it can be reprogrammed into many different tissue-specific cell types and, even so, their genome can be edited, making them an excellent model for linking phenotype to genotype. The emerging genome-editing technology of CRISPR-Cas9 (clustered regularly interspaced short palindromic repeats and CRISPR-associated protein 9) [192], which is more efficient, faster and cheaper than previous editing tools, has been used to efficiently modify endogenous genes and organisms, leading to further opportunity to generate genetically defined disease cell-based assay models [193].

Another trend to obtain more physiological disease models is the use of 3D cell cultures. Culturing cells in 3D environments promotes the formation of multicellular tissues with proper cell–cell and cell–matrix interactions necessary for full functionality. Natural and synthetic biomaterials and nanomaterials are utilized to build 3D cultures, also designated as spheroids, providing a robust architecture in 96- and 384-well format. This technology is being successfully applied in cancer models [194], although the culture medium and the materials used still need to be improved. Coupled with 3D cultures are the high-content imaging systems which have entailed huge advances in microscopy and image-informatics solutions [195]. Image acquisition using robotic fluorescent microscopy and automated image analysis has become an essential tool in early drug discovery programs. High-content cellular imaging has increasingly met the challenges of high-throughput needs and facilitates the integration of disease-relevant models and screens at early stages of the drug discovery process [196]. Together with quantitative fluorescence readouts in cell-based assays, non-invasive, label-free imaging techniques have recently emerged which satisfy the requirements of minimal cell manipulation, such as light sheet fluorescence microscopy (LSFM), that enable the analyses of many samples [197].

One step further is the organ-on-a-chip that is still under development. This technology is essentially a miniaturized microfluidic perfusion system which allows long-term in vitro growth of primary cells and tissues in a format viable for scaling up for high-throughput discovery campaigns. These systems model the complex tissue microenvironment and communication, reproducing in vivo tissue and organ functionality. One example reported by Maschmeyer et al. [198] is a four organ-chip system that mimics human liver, skin, intestines and kidneys. Furthermore, the use of microfluidic perfusion chambers in these systems permits the homeostatic function of the organ as if it were the blood flow, supplying nutrients and discharging catabolic metabolites [199].

In conclusion, great advances have been made in technologies to enable more precise and physiological models of diseases, increasing automatization, throughput and data management. Nonetheless, knowledge of the molecular basis of untreated diseases, new targets, the functional biological and physiological-based assay systems, and more predictive in vitro models are needed to achieve greater clinical relevance of the drugs discovered.

## Figures and Tables

**Table 1:** Different screening methods and their respective advantages and disadvantages.

**Method**	
**Advantages**	**Disadvantages**
**Antibacterial and antifungal screening**	
***Agar disk-diffusion method and variations***	
Simple, low cost High performance	Only qualitative results Not all fastidious bacteria can be tested
***Poisoned food method***	
Useful for evaluating antifungal effects Quantitative and qualitative results	Poor performance
***Thin-layer chromatography (TLC)-bioautography***	
Simple, low cost Suitable for bioactivity-guided fractionation	Poor efficiency for water-insoluble compounds
***Dilution method***	
Easy interpretation Quantitative results, suitable for MIC calculation Appropriated for fastidious or non-fastidious bacteria, yeast and filamentous fungi	Labor-intensive and time consuming Poor efficiency for water-insoluble compounds
***Time-kill test***	
Suitable for determining synergism or antagonism between bactericidal or fungicidal drugs Useful for determining the time- and concentration-dependent antimicrobial effect	Interlaboratory variability Labor-intensive and time consuming
***ATP bioluminescence assay***	
Fast, especially for antimycobacterial Quantitative results Suitable for testing in vivo	Expensive technique Requires specialized equipment
***Flow cytometry***	
Provide more information: detect antimicrobial resistance and target cell damage Fast	Requires specialized equipment
**Antibiofilm and antiquorum-sensing screening**	
***Colorimetric-based assays***	
Useful to assess the total biomass within a biofilm Highly accurate for large amounts of biofilm	Indirect measurement High detection limit No differentiation between dead and live cells
***Laser confocal microscopy***	
Direct measurement of biofilms 3D representation of biofilms	Fluorophores are required Reporter molecules are limited Fluorophores interference with biofilm Auto-fluorescence might mask fluorophores’ signal
***Disc diffusion assay***	
Simple, low cost Efficient	Qualitative results only Requires specific indicator strains
***Flask incubation assay***	
Simple, low cost Quantitative results	Requires specific indicator strains Indirect measurement
***Quorum quenching assay***	
Simple, low cost Efficient	Qualitative results Requires specific indicator mutated strains
**Anti-tropical diseases screening**	
***Kinetoplastid parasites***	
***Target-based screening***	
High performance assays (HTS)	Very few fully validated drug targets Additional screening is needed for avoiding off-target effects
***Phenotypic screening***	
High performance assays (HTS) and high-content imaging (HCS) in some parasite stages	Complex life cycles challenging to reproduce in laboratory Effectiveness in one parasitic stage does not guarantee the in vivo effect
***Helminths***	
***Target-based screening***	
High performance assays (HTS)	Very few fully validated drug targets Additional screening is needed for avoiding off-target effects
***Phenotypic screening***	
Use of *C. elegans* (nonparasitic specie) as model High-content imaging (HCS) in larvae	Few screening campaigns to date Complex life cycles challenging to reproduce in laboratory Effectiveness in one parasitic stage does not guarantee the in vivo effect
***Malaria***	
***Target-based screening***	
High performance assays (HTS)	Very few fully validated drug targets Additional screening is needed for avoiding off-target effects
***Phenotypic screening***	
Different methods developed for the different stages of life cycle: asexual erythrocytic-stage, liver stage, gametocyte Improvement in more physiologic in vitro human liver platforms High-content imaging techniques	Complex life cycles challenging to reproduce in laboratory Effectiveness in one parasitic stage does not guarantee the in vivo effect
**Anticancer screening**	
***Stained viable cells assay***	
Less expensive than other anticancer screening methods Quantitative results that are independent of the dye enzymatic conversion	Indirect measurement
***Dye exclusion assay***	
Inexpensive	Indirect measurement Time consuming User biased Low precision
***Methods based on metabolic activity***	
Suitable for HTS (MTT assays) Not cytotoxic for cells (Resazurin assay) High sensitivity (ATP content assay) Quantitative results	Indirect measurement Labor-intensive and time consuming
***Protease viability marker assay***	
Can be used in multiplex High sensitivity Not toxic Quantitative results	Indirect measurement
***Clonogenic cell survival assay***	
Highly precise results Quantitative results	Time consuming Only applicable to tumour cells that grow in culture
***DNA synthesis cell proliferation assay***	
Highly accurate and reliable Suitable for HTS Quantitative results	Use of radioactive labels Time-consuming protocol
**Neuroprotectors screening**	
***Stress reduction assays***	
Allows mimics in vitro some features of neurodegenerative diseases Suitable for the combination of target-based and phenotypic screening High performance assays (HTS) and high-content imaging (HCS)	Too much simplification of the diseases
***Neuroprotection assays***	
Allows mimics in vitro some features of neurodegenerative diseases Suitable for the combination of target-based and phenotypic screening High performance assays (HTS) and high-content imaging (HCS)	Too much simplification of the diseases
***Regeneration assays***	
More physiologic cellular models High-content imaging (HCS)	Challenging techniques and specialized equipment
